# Unveiling the complexity: assessing models describing the structure and function of the nuclear pore complex

**DOI:** 10.3389/fcell.2023.1245939

**Published:** 2023-10-09

**Authors:** Coby Rush, Zecheng Jiang, Mark Tingey, Fiona Feng, Weidong Yang

**Affiliations:** Department of Biology, Temple University, Philadelphia, PA, United States

**Keywords:** nucleocytoplasmic transport, super-resolution light microscopy, nuclear pore complex, transmembrane proteins, intrinsically disordered protein

## Abstract

The nuclear pore complex (NPC) serves as a pivotal subcellular structure, acting as a gateway that orchestrates nucleocytoplasmic transport through a selectively permeable barrier. Nucleoporins (Nups), particularly those containing phenylalanine–glycine (FG) motifs, play indispensable roles within this barrier. Recent advancements in technology have significantly deepened our understanding of the NPC's architecture and operational intricacies, owing to comprehensive investigations. Nevertheless, the conspicuous presence of intrinsically disordered regions within FG-Nups continues to present a formidable challenge to conventional static characterization techniques. Historically, a multitude of strategies have been employed to unravel the intricate organization and behavior of FG-Nups within the NPC. These endeavors have given rise to multiple models that strive to elucidate the structural layout and functional significance of FG-Nups. Within this exhaustive review, we present a comprehensive overview of these prominent models, underscoring their proposed dynamic and structural attributes, supported by pertinent research. Through a comparative analysis, we endeavor to shed light on the distinct characteristics and contributions inherent in each model. Simultaneously, it remains crucial to acknowledge the scarcity of unequivocal validation for any of these models, as substantiated by empirical evidence.

## 1 Introduction

### 1.1 The nuclear pore complex

#### 1.1.1 Subregions

Nuclear pore complexes (NPCs) are intricate protein assemblies located on the nuclear envelope (NE) that facilitate the transport of macromolecules between the cytoplasm and nucleus. NPCs stand out from other protein complexes due to their remarkable size and complexity, with a molecular weight of approximately 110 MDa in humans (Kosinski et al., 2016; Lin et al., 2016; Lin and Hoelz, 2019). This complexity is conserved across different species and primarily arises from the presence of specialized proteins known as nucleoporins (Nups). Nups can be classified into three main groups based on their localization and functions: transmembrane Nups, which are integrated into the NE structure; central scaffold Nups, providing structural support; and phenylalanine–glycine (FG)-Nups, which constitute the selective barrier within NPCs (Beck and Hurt, 2017; Bindra and Mishra, 2021).

**FIGURE 1:**
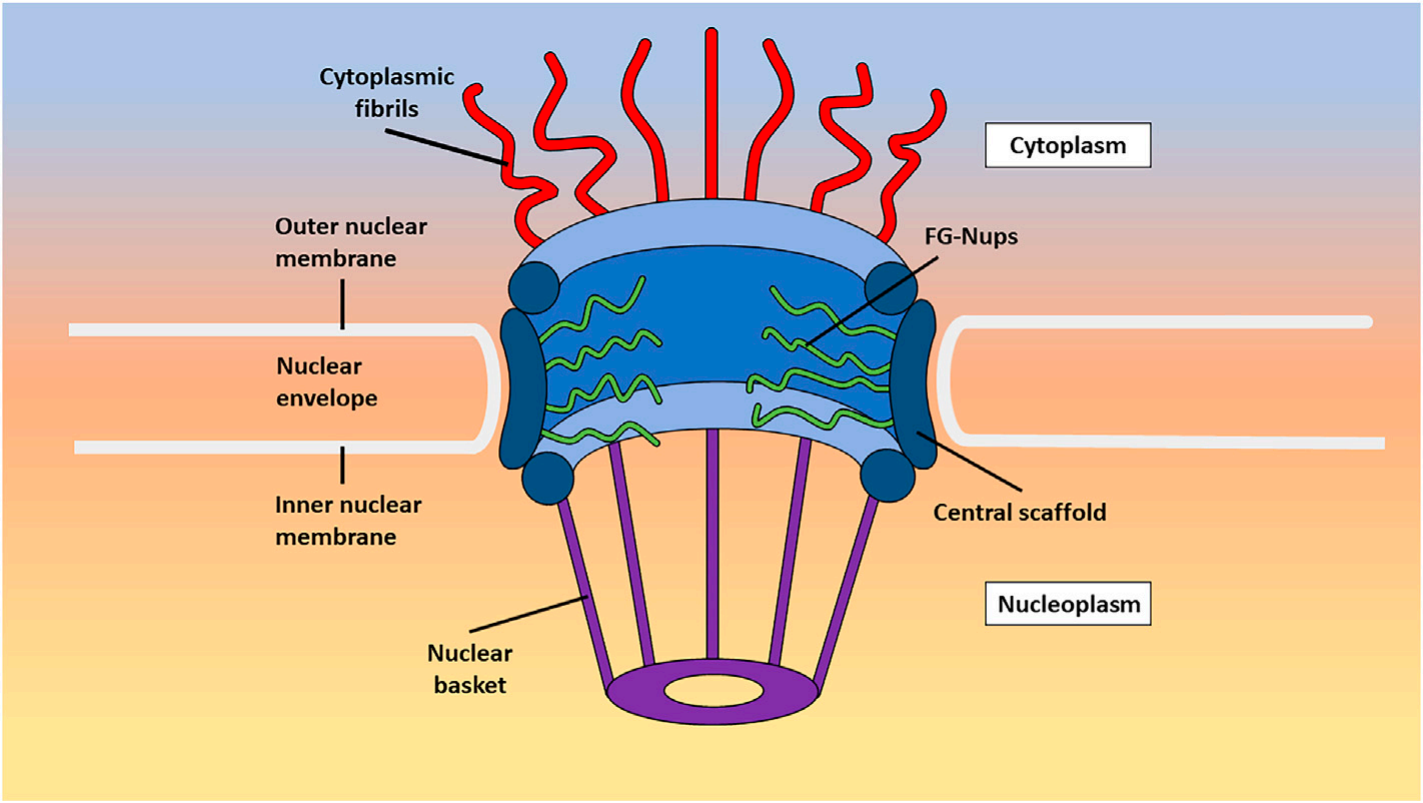
An annotated image detailing the subregions of the NPC. Shown here is a stylized image of the different subregions of the NPC as they exist in live cells including the cytoplasmic fibrils, central channel, and nuclear basket.

NPCs comprise three primary subregions: the cytoplasmic fibrils, central scaffold, and nuclear basket (Figure 1). The cytoplasmic fibrils, as the name implies, are located on the cytoplasmic side of the NPC and extend approximately 50 nm into the cytoplasm. The central scaffold connects the cytoplasmic fibrils to the nuclear basket and consists of protomer spokes that exhibit octameric symmetry. These spokes interconnect to form the inner pore ring with a diameter of approximately 50 nm, the outer pore ring with a diameter of approximately 120 nm, and the luminal ring. Within the central scaffold, the central channel serves as the pathway for macromolecular transport. The selectively permeable barrier of the NPC resides within the central channel and is composed of FG-Nups, which regulate the passage of molecules larger than the passive diffusion limit of approximately 40 kDa (Li et al., 2016). Lastly, the nuclear basket is situated on the nuclear side of the NPC and spans approximately 75 nm in length. It comprises eight fibrils arranged in a basket-like structure and facilitates the docking and export of macromolecules through the central channel (Stoffler et al., 2003; Maimon et al., 2012; Li et al., 2021; Tingey et al., 2022).

#### 1.1.2 Nucleoporins

There are three main types of Nups found in the NPC: transmembrane Nups, structural Nups, and FG-Nups. Transmembrane Nups serve as anchors by connecting the NPC to the nuclear envelope through transmembrane helices (Hoelz et al., 2011). They also interact with non-transmembrane Nups to ensure the stable assembly of NPCs, playing a crucial role in maintaining the normal structure and function of the NPCs (Shevelyov, 2020). The absence of transmembrane Nups can lead to assembly errors, such as mislocation or abnormal shape of NPCs (Madrid et al., 2006; Shevelyov, 2020).

Structural Nups, also known as scaffold Nups, form the structural framework of the NPC. They are primarily located between the nuclear and cytoplasmic groups of Nups and contribute to anchoring them together through α-solenoid and β-propeller domains (Devos et al., 2006). These Nups exhibit a pattern of eight-fold rotational symmetry, although occasional nine-fold rotational symmetry is observed. One exception is POM 121 in metazoans, which functions as both a transmembrane protein and an FG-Nup. Transmembrane and structural Nups adopt secondary structures such as β-propeller and α-solenoid motifs, while disordered FG-Nups lack typical secondary and tertiary structures.

FG-Nups derive their name from the presence of FG tandem repeats. Approximately one-third of the approximately 30 Nups in the NPC contain multiple FG-repeat domains. Assuming an average of two copies of each FG-Nup per spoke in the eight-fold symmetrical NPC, there are over 200 FG-Nups in a single NPC. These FG domains extend to the cytoplasmic filaments and the nuclear basket, encompassing approximately 4,000 FG domains available to form an interior structure inside the central channel. The FG domains are natively disordered and lack an obvious secondary structure. Although some non-FG-Nups are present in the NPC channel and interact with FG-Nups, it is widely accepted that the selective barrier is primarily formed by the FG domains of FG-Nups, permitting the transport of small molecules while blocking the passive transport of larger macromolecules. There are three major subtypes of FG-Nups classified by their particular repeating sequences. These include FxFG (phenylalanine-x-phenylalanine–glycine), GLFG (glycine–leucine–phenylalanine–glycine), and xxFG (x-x-phenylalanine–glycine). Additionally, there are minor types such as PSFG (proline–serine–phenylalanine–glycine), SAFG (serine–alanine-phenylalanine–glycine), and VFG (valine–phenylalanine–glycine) (Rout and Wente, 1994). These subgroups maintain different biochemical and biophysical properties, caused by variation in traits such as hydrophobicity and charge (Fiserova et al., 2014). Studies have demonstrated the crucial role of the bidirectional selective barrier formed by FG-Nups in various cellular processes, including mitosis, DNA repair, regulation of gene expression, and protein synthesis (De Souza and Osmani, 2007; Tavolieri et al., 2019; Ashkenazy-Titelman et al., 2020; Akey et al., 2022; Coyne and Rothstein, 2022).

#### 1.1.3 Intrinsically disordered FG-Nups

Much of the study of proteins is centered around structurally consistent proteins, in which a particular series of amino acids makes up the primary sequence. Within a native environment, the composition of the amino acid sequence causes the protein to conform to a specific, energetically favorable structure. Even these stable protein structures tend to move for several reasons, including different environmental conditions such as denaturing and directed changes through substrate interactions, allosteric effectors, or covalent modification. Interestingly, there are also small conformational adjustments known as “breathing,” where the protein undergoes minute conformational changes. Even though these structural changes are possible, many proteins are still believed to have at least a semi-rigid conformation (Kossiakoff, 1982; Bu and Callaway, 2011).

This description of proteins is limited, however, and ignores a significant type of proteins that do not have a particular native confirmation referred to as intrinsically disordered proteins (IDPs) (Uversky, 2013; Oldfield and Dunker, 2014; Uversky, 2014; Uversky, 2020; Vovk and Zilman, 2023). IDPs contain both structured and intrinsically disordered regions (IDRs) and represent a relatively recent expansion of our understanding of the protein structure. While IDRs have various primary structures, there are some common patterns found within these regions. They often contain elevated levels of proline and glycine, which are known to reduce the ability of polypeptides to form ordered structures. Conversely, the amino acids cysteine and asparagine, which are known to promote the formation of ordered structures, tend to be absent in IDRs. In general, disordered proteins tend to have simple primary structures, with characteristically repetitive amino acid residues. In addition, IDPs tend to have low hydrophobicity and high net charge, meaning there are fewer hydrophobic interactions to drive compaction and more charge–charge repulsion to drive disorder. There are a few separate roles that IDPs play in cell biology, with one of the major roles being cell signaling, in which the disordered nature of these proteins allows for more diverse binding to signals (Zhou et al., 2018; Bondos et al., 2021). Another extremely crucial role for these IDPs to play in the cell, and the one that will be the primary focus of this review, is within the NPC as FG-Nups.

Interestingly, while FG-Nups contain high levels of glycine, one of the most common amino acids found in IDRs, they are also rich in phenylalanine, which is one of the most hydrophobic amino acids. This presents the seemingly contradicting nature of these proteins, which is a recurrent theme throughout this review. While FG-Nups are intrinsically disordered enough to be flexible and allow the cargo to pass through the NPC, they also must have strong enough interactions, and therefore enough structure, so that cargo should not aimlessly leak back and forth between the cytoplasm and nucleoplasm. As discussed further in this review, different models of the NPC emphasize different interactions between FG-Nups to explain their behaviors.

#### 1.1.4 Passive and facilitated transport

The presence of FG-Nups in the NPCs establishes a selective barrier that governs the transport of macromolecules through two major mechanisms: passive diffusion and facilitated diffusion. Passive diffusion through the NPC involves the movement of small molecules down their concentration gradient, without the need for direct energy input (Samudram et al., 2016; Timney et al., 2016). Small molecules with a molecular weight below 40–60 kDa can freely diffuse through the pore (Paine and Feldherr, 1972; Peters, 1983), traversing the NE bidirectionally. This passive diffusion mechanism enables the movement of small molecules between the nucleus and the cytoplasm without requiring active transport processes (Samudram et al., 2016). However, it is worth noting that the two-way selection barrier of the NPC is not a rigid barrier. Early studies in the 1970s and 1980s indicated that the NPC had some form of passive diffusion channel with a radius of approximately 4.5–5.9 nm (Paine and Feldherr, 1972; Peters, 1983), which was generally assumed to be rigid with a defined size threshold (Ribbeck and Görlich, 2001; Mohr et al., 2009; Ma et al., 2012). However, follow-up studies and observations show that the threshold for passive diffusion through the NPC is not rigid, allowing for the passive diffusion of larger molecules (Wang and Brattain, 2007; Kirli et al., 2015; Popken et al., 2015). It is certain that several FG-Nups are required to establish and regulate passive diffusion, a precise description of which is still under discussion (Shulga et al., 2000).

Particles with masses above the passive diffusion limit, such as large proteins and mRNPs, require facilitated transport mediated by transport receptors (TRs), also known as karyopherins (Ashkenazy-Titelman et al., 2020). These TRs recognize nuclear localization signals (NLSs) or nuclear export signals (NESs) on cargo (Görlich and Kutay, 1999; Kuersten et al., 2001; Weis, 2002). Typically, TRs transport cargo unidirectionally, classifying them as importins or exportins based on the direction of translocation, although some function bidirectionally (Baade and Kehlenbach, 2019). TRs facilitate transport through the NPC via multivalent interactions with FG-Nups in the central channel (Kapinos et al., 2014). The specific mechanisms of NPC gating are not fully understood, but interactions between TRs and FG-Nups involve hydrophobic grooves on HEAT repeats (Cingolani et al., 1999; Vetter et al., 1999), which are common motifs of two short α-helices often found on TRs (Yoshimura and Hirano, 2016). These interactions must strike a balance between associations strong enough to facilitate transport and weak enough to promote rapid translocation (Pemberton and Paschal, 2005). Facilitated transport through the NPC relies on specific TRs that recognize and bind to cargo molecules, such as proteins or RNA, and is primarily powered by the Ran GTPase cycle (Cole and Hammell, 1998; Moore, 1998; Lyman et al., 2002; Isgro and Schulten, 2005). Ran is a 25-kDa protein that transitions between GTP- and GDP-bound states (Moore, 1998). Prior to nuclear export, the exportin binds to both the cargo and Ran–GTP, and after entering the cytoplasm, the GTP is hydrolyzed, forming Ran–GDP and releasing the cargo from the exportin. Conversely, after the facilitated import of cargo, Ran–GTP in the nucleus binds to the importin, dissociating it from the cargo and terminating the transit process (Moore, 1998; Strambio-De-Castillia et al., 2010). In general, facilitated transport through the NPC is tightly regulated, with selectivity and directionality controlled by TRs, the RanGTP cycle, and interactions with Nups (Rexach and Blobel, 1995; Görlich et al., 1996).

### 1.2 Methods to resolve the structure and function of the NPC

#### 1.2.1 *In vitro* analysis

Due to the intrinsically disordered nature of FG-Nups, they can be equally as fascinating as they are challenging for researchers of cell biology. Furthermore, the intrinsically disordered nature has complicated resolving the complete structure of the NPC. Techniques including X-ray crystallography, electron microscopy (EM), and electron tomography (ET) have brought the structure of the NPC scaffolds to atomic-scale resolution; however, they were unsuccessful when trying to resolve the dynamic nature of the interior of the NPC (Brohawn et al., 2009; Huang et al., 2023). Because these techniques cannot directly provide information about movement and can only provide a static structure, they leave our understanding of the NPC incomplete (Davis et al., 2003; Renaud et al., 2018). Older EM data have presented a structural barrier residing within the center of the NPC known as the “central plug,” and it was thought that passaging cargo had to either move around or interact with this plug (Talcott and Moore, 1999). This finding generated what is known as the “plug model” of the NPC, which is further explored in this review; however, the composition of the plug and the apparent mobility relative to the scaffold of the NPC were unclear (Stoffler et al., 2003). More recent models produced by these techniques tend to just omit the inside of the pore, leaving approximately a 60-nm-diameter gap where the FG-Nups would otherwise be displayed (Brohawn et al., 2009; Von Appen et al., 2015; Schuller et al., 2021; Tai et al., 2023). This is not just a gap in depictions of the pore but a gap in our knowledge about the pore because even though it is now certain that the pore is filled with FG-Nups, it remains uncertain exactly how they are composed.

Nuclear magnetic resonance (NMR) spectroscopy is often used to determine protein structures that are more flexible or undergo conformational change, and so it has been applied to gain structural information about IDPs. However, this is typically performed *in vitro* and may not account for the complex native cellular environment of that protein. In addition, despite being a valuable tool for evaluating IDPs and having been used to help study the structure of individual Nups (Hough et al., 2015; Milles et al., 2015; Tai et al., 2023), NMR is not suited to studying the entirety of FG-Nups within the NPC. Rather, NMR is primarily useful for smaller, less complicated systems.

#### 1.2.2 *In silico* analysis

One of the more recently developed methods of studying the behaviors of FG-Nups, and intrinsically disordered proteins in general, is computer simulation experiments (Moussavi-Baygi et al., 2011; Best, 2017). Using a simulation to model the entirety of the NPC in its native environment does not seem feasible with the current technology, and there are ways in which the intricacy of the complex must be reduced to perform these studies (Mincer and Simon, 2011; Moussavi-Baygi et al., 2011; Azimi and Mofrad, 2013). A full simulation would need to include thousands of FG-Nups, facilitated and passive diffusion of many different particles over the timescale of milliseconds, the presence of TRs in the pore, decorated Nups, and the consideration that the scaffold itself is somewhat flexible. While it may be a possibility in the future to simulate the entirety of the NPC without any simplification, any conclusions determined by simulation, if not also confirmed with live-cell measurements, cannot be fully accepted as accurate. Artificial intelligence models such as AlphaFold, which are designed to predict the protein structure, are on the rise and have assisted in solving the scaffold of the NPC (Fontana et al., 2022). However, these models struggle to provide information about IDPs as a result of their disordered nature and, therefore, are not currently fully applicable to FG-Nups (Ruff and Pappu, 2021; Mosalaganti et al., 2022).

Despite the wide array of techniques used to investigate the structure of the NPC, there are still many questions about the structure of the pore and behavior during nucleocytoplasmic transport. Ultimately, no single technique is sufficient to fully investigate this behemoth of a cellular structure, and most studies performed with the goal of resolving the complex have been multidisciplinary and multimodal. An example of this is a study performed on the transport of HIV-1 capsids through the NPC, using a combination of correlative light and EM (CLEM) and cryo-FIB milling to gain dynamic, highly resolved, *in situ* information about this event (Zila et al., 2021). CLEM is a technique that combines the resolution abilities of EM with the dynamic ability of fluorescent microscopy (Van Rijnsoever et al., 2008; Haraguchi et al., 2022).

#### 1.2.3 Native environment

One of the major concerns when studying IDPs *in vitro* is that it may not account for the effect of the levels of crowding that occur within the cell. There are two ways to overcome this: one is by trying to replicate a cellular environment *in vitro* by adding various crowding agents and the second is to perform the study either *in situ* or, ideally, *in vivo* and study the structure as it stands in its cellular environment. Crowding inside the cell could have multiple effects, and it may be the case that due to crowding, IDPs end up becoming more ordered because of interactions between themselves and with the surrounding proteins. Considering FG-Nups, the presence of so many copies packed within a single NPC may cause crowding-induced structural stability (Kuznetsova et al., 2014). Additionally, it has been shown that TRs reside inside the NPC, which may then cause the FG-Nups to hold different conformations than they would in the absence of these TRs (Schoch et al., 2012; Kapinos et al., 2014; Wagner et al., 2015).

Even though cryo-EM cannot study the NPC in a live-cell environment, there is a modification to the technique known as cryo-focused ion beam (cryo-FIB) milling that allows for *in situ* analysis of the nucleus (Villa et al., 2017) and specifically the NPC in its native environment (Mahamid et al., 2016; Mosalaganti et al., 2018; Allegretti et al., 2020; Schuller et al., 2021). The general principle of FIB milling is that, after preparing the sample for EM, beams of ions bombard the target surface, ejecting atoms from the sample in a process known as sputtering. This beam is used to scan the sample, layer by layer, until only the desired target layer remains, permitting the EM analysis of samples that would otherwise be too thick (Schertel et al., 2013; Schaffer et al., 2019). These studies have found that human NPCs can be substantially larger in diameter than previously understood based on previous models (Schuller et al., 2021) and that in fact, the pore can dilate during transport and condense during periods of low cellular energy (Zimmerli et al., 2021). These findings contribute to what has been described as the “dilation model” of the NPC, which is explored later in this review. While this is an improvement over traditional cryo-ET in allowing for observations in the native cellular environment, cryo-FIB still cannot provide dynamic, *in vivo* information about the NPC and, therefore, cannot create a complete picture of the complex.

Atomic force microscopy (AFM) has been used to probe the behaviors of proteins and has specifically been applied to studying the behaviors of the NPC (Oberleithner et al., 2000; Shahin et al., 2005; Vial et al., 2023). Essentially, this technique involves a nanometer-scale tip that can physically probe surfaces and measure both the topography of a sample and force measurements. AFM has been used to study the behaviors of Nup153 *in vitro* (Lim et al., 2007); however, it does not possess sufficient spatiotemporal resolution to study individual Nups within the NPC. Fortunately, the development of high-speed AFM (HS-AFM) has improved the technique with more delicate, faster tapping, which decreases invasiveness, and rapid scanning to provide temporal resolutions of approximately 100 ms (Sakiyama et al., 2016). HS-AFM has been used to study the NPC in fixed *Xenopus laevis* cells, and more recently, in live human colorectal cancer cells (Mohamed et al., 2017). Unfortunately, the temporal resolution of HS-AFM is still not sufficient to measure the dynamics of transport processes, which are on the scale of milliseconds, and the spatial resolution still cannot compete with super-resolution light microscopy. In addition, despite advancements in the delicacy of the technique, AFM is invasive by nature and, therefore, measurements will be impacted by the measuring device itself. Combining AFM with other higher-resolution microscopy techniques such as single-molecule localization microscopy (SMLM) produces more robust methods of analyzing the NPC (Vial et al., 2023).

Another powerful class of techniques that allows for the investigation of the NPC, not only in its native environment but also *in vivo*, is super-resolution fluorescence microscopy. Although other light microscopy techniques such as confocal microscopy can be used to study the NPC (Kirli et al., 2015), in order to reach the resolution required to detect and track single molecules, super-resolution microscopy must be utilized, including methods such as structured illumination microscopy (SIM) (Chatel et al., 2012; Coyne et al., 2021), stimulated emission depletion (STED) microscopy (Wurm et al., 2012; Göttfert et al., 2013), and SMLM (Schlichthaerle et al., 2019; Sabinina et al., 2021; Andronov et al., 2022; Vial et al., 2023). Cutting-edge high-speed SMLM microscopy techniques offer unparalleled insights into the transport kinetics and structural dynamics of Nups and transported cargo within living cells (Hüve et al., 2008; Lowe et al., 2010; Ma and Yang, 2010; Goryaynov et al., 2012; Ma et al., 2012; Atkinson et al., 2013; Ma et al., 2016; Junod et al., 2020; Mudumbi et al., 2020; Pulupa et al., 2020; Li et al., 2021). Moreover, when coupled with post-localization algorithms, one of the methods has recently enabled the acquisition of three-dimensional super-resolution structural and dynamic information within the sub-micrometer live NPCs (Ma et al., 2016; Mudumbi et al., 2020; Li et al., 2021).

Due to the abundance of sometimes conflicting information concerning NPC behaviors, the complete resolution of its structure and function remains elusive. This situation has paved the way for the emergence of diverse models aimed at elucidating pore mechanisms. This review offers an encompassing view of the various models that have emerged, combining theoretical and experimental findings with the aspiration of illuminating the true intricacies within the NPC.

## 2 Models of the NPC and nucleocytoplasmic transport

### 2.1 Plug model

**FIGURE 2:**
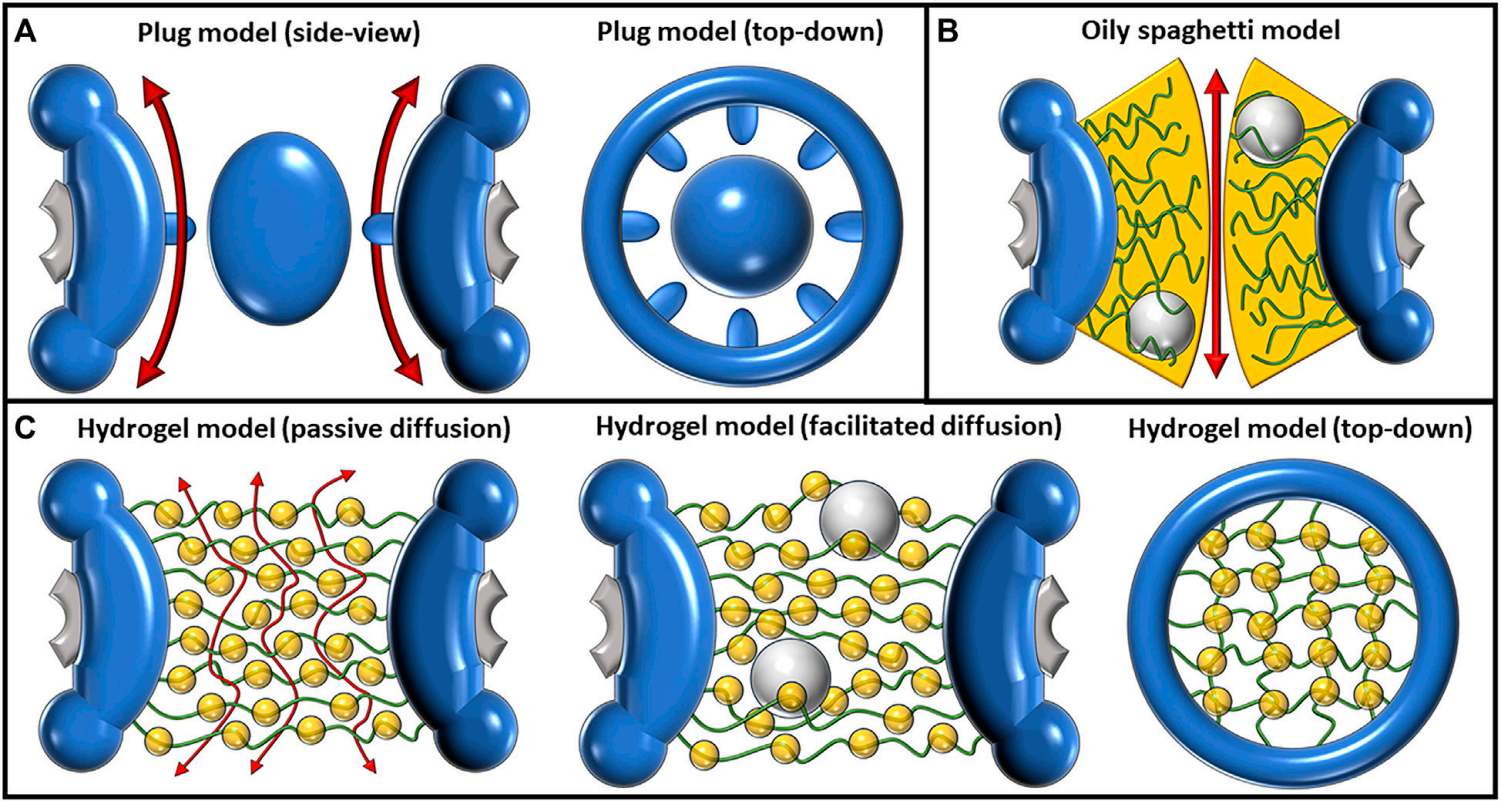
Depictions of the plug, oily spaghetti, and hydrogel models and their proposed methods of transport. **(A)** The plug model shown from a side view (left) and top-down view (right). The plug model depicts a large, spheroidal mass in the center of the pore. The exact composition of this large mass, termed the “central plug,” is unknown. It was predicted that passive diffusion had to move around the plug, as shown by the double-sided red arrows flanking the plug (left). The eightfold symmetrical scaffold (right) has also been an observed feature of this model. **(B)** The oily spaghetti model predicts that facilitated diffusion occurs via random movement of FG-Nups which transiently interact with large cargo. The region of transient interaction is shown in yellow. Passive diffusion of small particles may proceed without requiring interactions with the FG-Nups, shown by the double-sided red arrow. **(C)** The hydrogel/selective phase model as shown from a side view during passive transport (left), facilitated transport (middle), and from a top-down view without transport occurring (right). The regular hydrophobic interactions between FG-Nups in this model create a meshwork (right) where small particles can passively transport between the gaps (left). Large particles must disrupt these hydrophobic interactions in order to transit and are facilitated by their own transient interactions with the FG-Nups.

The central plug, also known as the central transporter, within the NPC, is a relatively early development in the quest to resolve the inner structure of the NPC. First described by Unwin and Milligan in 1982 as a result of a cryo-EM study on *Xenopus* oocytes, the central plug was shown to be a large, spheroidal particle residing in the very center of some of the pores, possessing a diameter of approximately 35 nm (Unwin and Milligan, 1982) (Figure 2A). This structure has been both mysterious and controversial, leading to much debate about its composition and function. While the exact nature of the plug has been unclear, the consensus, however, has been that it is a dynamic structure that presents itself only under certain conditions (Reichelt et al., 1990; Beck et al., 2004; Schwartz, 2005; Lim and Fahrenkrog, 2006). As a result, more recent studies using cryo-EM or similar techniques tend to omit the plug and present the structure of the scaffold by itself (Eibauer et al., 2015; Von Appen et al., 2015; Zhang et al., 2020; Zila et al., 2021). Speculation has arisen suggesting that the presence of plugs could potentially stem from the entrapment of sizable cargo while in transit during imaging procedures (Stoffler et al., 2003). However, a study by Li et al. employed cryo-electron microscopy (cryo-EM) for visualizing pre-60S particles ensnared within yeast NPCs. Intriguingly, these substantial particles exhibit a predilection for tracing the periphery of the pore structure, veering away from a central trajectory. This observation holds the promise of tempering the plausibility of pre-60S particles being the probable origin of the observed plug phenomenon (Li et al., 2023).

A possible mechanism of transport utilizing the plug was described by Talcott and Moore (1999). According to their description, the central plug acts as a barrier that prevents passive diffusion from occurring through the middle of the central channel of the pore, forcing small particles instead to traverse peripherally. This passive diffusion occurs through eight symmetric channels that are each ∼10 nm in diameter and surround the center of the pore. Due to filling nearly the entire interior of the central channel, the plug also acts to control facilitated diffusion, blocking the transit of large particles and providing some form of substrate or channel with which signal-mediated import and export can occur. In this model, facilitated transport would cause reversible displacement or deformation of the plug, which may assist in explaining why the pore has been observed to vary between images of individual pores. However, the model does not provide a plausible explanation for whether the shape change requires the NPC itself to use energy or whether the NPC’s structure is flexible enough to allow sufficiently large molecules to pass through. Thus, additional research is needed to elucidate the mechanisms by which the NPC undergoes shape changes and facilitates the transport of larger molecules as well as to determine the energy requirements associated with these processes.

Beck et al. (2004), while studying the NPCs of *Dictyostelium discoideum* using cryo-ET, not only showed the variability of the central plug but also provided some new structural insights (Beck et al., 2004). Their findings indicate that the plug is not a single spheroidal density, but rather two overlapping densities. The smaller one is in the same plane as the cytoplasmic filaments, and the larger one is closer to the center, slightly biased toward the nuclear side of the pore. In a 2016 paper, Sakiyama et al. utilized HS-AFM to analyze the spatiotemporal dynamics of *X. laevis* NPCs (Sakiyama et al., 2016). It was found that within this population of NPCs, approximately 40% contained a “plug-like” feature, which they have stated is likely cargo trapped in the pore. In addition, they characterize a “central plug/transporter” (CP/T) which is also a centrally located object; however, it is distinct from the “plug-like” features. Analyzing their dynamic data by plotting the mean squared displacement (MSD) in the *z*-direction over time, they found that the FG-Nups inside the pore behave as tethered polypeptides, reaching MSD saturation. The z-diffusional limit was found to be approximately 0.9 nm for FG-Nups near the edge of the pore, while for those near the center, it was approximately 1.6 nm. The authors indicate that as a result of the increased mobility of FG-Nups closer to the center of the pore, the identity of the CP/T is likely to be the result of averaging this dynamic motion, creating what appears to be a static component. This conclusion was further explored in a 2020 paper by Mohamed et al. who also utilized HS-AFM to study the central plug, coming to a similar conclusion that the central plug is at least partially composed of FG-Nups (Mohamed et al., 2020). They described the behavior as FG-Nups forming transient “knots” among themselves in the center of the pore and found that the plugs in cancerous HCT116 cells had a greater extent of conformational dynamics than non-cancerous colon cells. The conception of the central plug architecture can be attributed to the inherent constraints of cryo-EM, primarily its necessity to arrest samples in a frozen state. The plug model was conceived as an initial endeavor to elucidate the inner workings of the NPC. Despite its vintage, the definitive nature of this putative plug element remains unknown. This model, although aging, has retained its utility as a foundational template, inspiring subsequent explorations that have spurred investigators to ponder profound queries concerning the NPC's composition, structure, and transport mechanisms. These inquiries have set the cornerstone for the evolution of more contemporary models, designed to unravel different facets of this intricate structure. As technology continues to progress, expanding our comprehension of the pore, the underpinning framework of the plug model has undergone refinement, harmonizing with fresh insights to engender novel questions, studies, conjectures, and models. The advent of dynamic, live-cell data holds the promise of potentially unveiling the elusive identity of the plug, finally unraveling a longstanding biological enigma.

### 2.2 Polymer brush model

**FIGURE 3:**
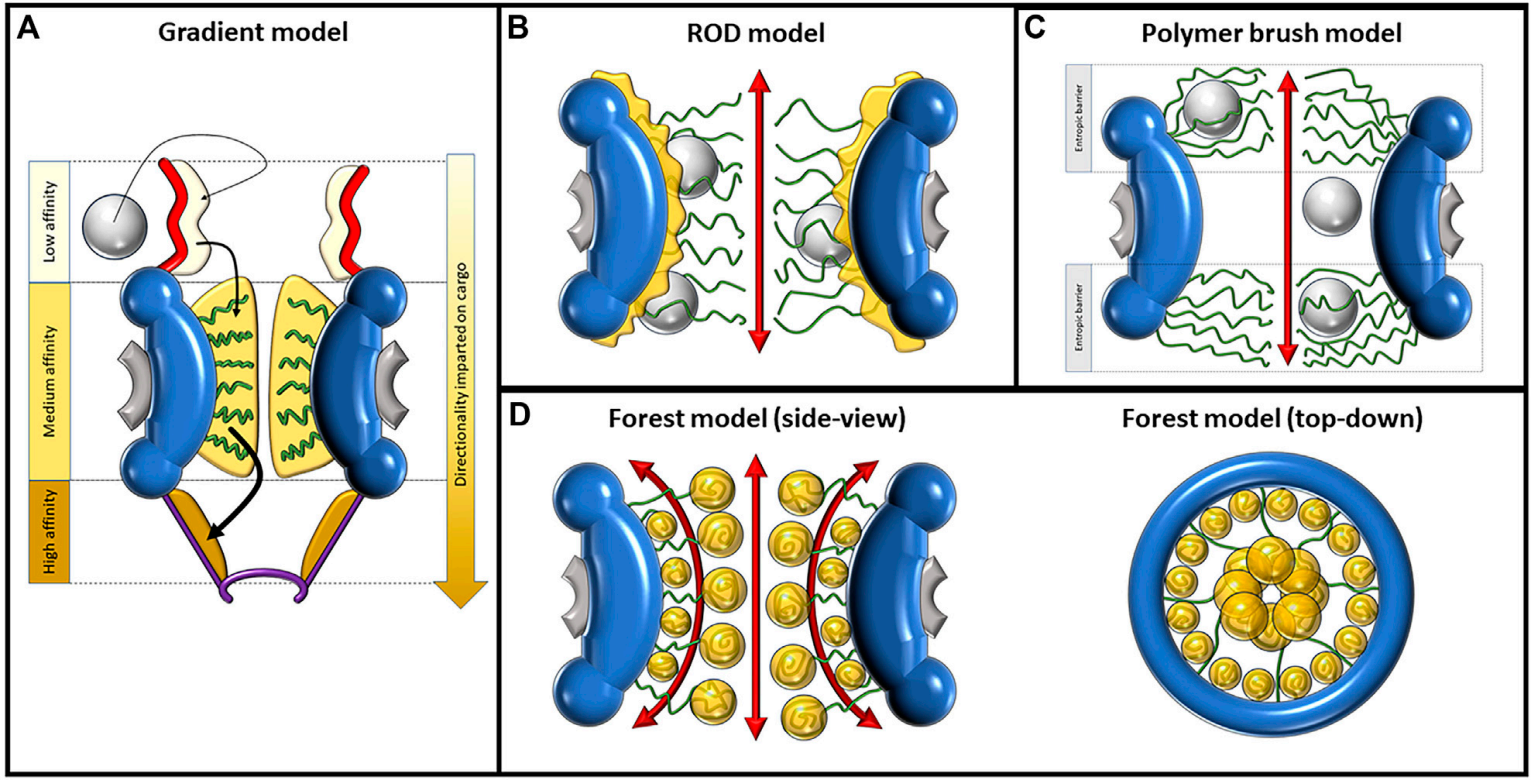
Depictions of the gradient, ROD, polymer brush/virtual gate, and forest model, displaying their proposed mechanisms of nucleocytoplasmic transport. **(A)** Mechanism of import as described by the gradient model. The direct and facilitated diffusion of the large cargo, depicted as a gray sphere, is caused by increasing levels of affinity to FG-Nups in three domains moving toward the nucleoplasm. In order of increasing affinity, these domains are the cytoplasmic fibrils, central channel, and nuclear basket. FG-Nups are shown as green curved lines. **(B)** The ROD model predicts two distinct transport routes for passive and facilitated diffusion. The route of passive diffusion, as shown by the double-sided red arrow, is directly through the center of the NPC. Facilitated diffusion occurs against the inner walls of the pore, where collapsed FG-Nups interact with large cargo, imparting a random 2D walk on the cargo. **(C)** The polymer brush/virtual gate model assumes that randomly fluctuating FG-Nups, repelled by electrostatic forces, create an entropic barrier on both openings of the pore. While small cargo can passively diffuse between this entropic barrier, large cargo must overcome it via interactions with FG-Nups. **(D)** The forest model shown from the side-view (left) and top-down view (right). Some FG-Nups take the form of “shrubs” with collapsed coils lining the inside walls of the pore, while the “trees” have longer disordered regions reaching into the center of the NPC, where their collapsed coil regions aggregate. The collapsed regions are dominated by hydrophobic interactions and the disordered regions by electrostatic interactions. This composition creates two distinct transport zones; the first is a route through the center of the pore and the second is made of multiple different routes more peripherally located.

The concept of the polymer brush model, also referred to as the virtual gating model, was initially introduced by Rout et al. in October 2003 (Rout et al., 2003). This model stands as an intuitive framework aimed at elucidating the mechanisms underlying the passive, swift, and discerning translocation of cargo facilitated by the FG-Nups. Rooted in biochemical and biophysical thermodynamics, the polymer brush model offers insights into the functionality of the NPC. According to the polymer brush model, the NPC’s central channel is envisaged as an entropic barrier. This barrier serves as the foundation for both mediated passive and facilitated transport through the pore (Shulga and Goldfarb, 2003). Broadly, the model seeks to expound upon the pore’s capacity to regulate the assortment of cargo that can traverse it, based on the principle of electrostatic repulsion. This inherent repulsion gives rise to the barrier-like feature, shaping the pore’s ability to selectively permit certain molecules while excluding others. In essence, the polymer brush model encapsulates the intricate dynamics of the NPC’s cargo transport, offering a cogent explanation rooted in fundamental biophysical principles. Entropy (S) refers to the number of ways the energetic motions of a macromolecule can be distributed. Within the cytosol, a macromolecule can move freely, resulting in high entropy. The central channel of the NPC restricts movement, leading to decreased entropy. Therefore, placing a macromolecule within the central tube comes with an entropic penalty, and a region densely populated with FG-Nups increases that cost by further restricting available diffusion space (Hoh, 1998). A macromolecule needs to enter a “transition state” to pass through the NPC. One way to achieve this is through affinity and binding to the FG-Nups in the NPC. Macromolecules need to bind NTRs to enter a “transition state” and pass through (Rout et al., 2003). Although the rope-like structure of FG-Nups may allow them to move aside to let macromolecules pass through, this also requires energy, so macromolecules that are not bound to NPCs have a very low probability of entering the “transition state,” and subsequently crossing the NE. In this model, cargo smaller than 30 nm can pass through the narrow channel protected by the FG-Nup barrier if they can afford to pay the entropic penalty. With the increase in size of a macromolecule, the entropic cost of passing through the central tube increases and the probability of passage decreases. Beyond a certain size, the probability of passage becomes negligible (Rout et al., 2003) (Figure 3C).

Furthermore, the polymer brush model explains translocation across the NPC using the Gibbs free energy (ΔG) of a system. Gibbs free energy is defined as the difference between the system’s change in enthalpy (ΔH) and the product of its temperature (T) and change in entropy (ΔS) (Eq. (1)). In a simplified consideration of the NPC system, the ΔS across the NE describes the entropic barrier of the NPC, while the ΔH describes the binding energy of macromolecules to the NPC. Passing through a physical barrier such as the pore temporarily reduces entropy, resulting in a positive ΔG and creating an energy barrier to activation, causing the process to be thermodynamically unfavorable (e.g., a nonbinding macromolecule attempting to cross the NPC). For translocation to occur, the ΔG must be decreased below the diffusion energy available to a macromolecule (kT). This can be achieved by using binding energy as compensation to decrease ΔH, flattening the energy landscape and reducing the activation energy of translocation across the NE. Binding also has an entropic term, but the sum of the binding and diffusion entropies can be canceled out by sufficient ΔH. In an optimal scenario, the binding and barrier energies are balanced, allowing a macromolecule to pass rapidly through the NPC with minimal hindrance.

∆G=∆H−T∆S. (1)

In the kinetics of binding, the authors note that using binding sites to overcome an entropic barrier has its drawbacks. A binding macromolecule spends time attached to its binding sites, which slows down its overall translocation rate across the NPC. If the process takes too long, translocation becomes excessively slow. Effective binding sites surrounding the central tube must have low enough affinities and high enough off-rates to enable the rapid passage of transport factors through the channel. High-affinity binding sites tend to exhibit low off-rates, which can retain bound macromolecules for an extended period, impeding their passage or even trapping them at the NPC (Rout et al., 2003). The authors propose that the NPC possesses numerous low-affinity binding sites, allowing the central channel to provide sufficient binding energy to lower the entropic barrier without compromising transport speed (Rout et al., 2003). In conclusion, the virtual gating model suggests an entropic barrier constituted by a highly dynamic and non-cohesive polymer brush of FG-Nups with weak FG–FG associations. Large TR–cargo complexes require enthalpic gain from hydrophobic TR–FG associations to overcome the entropic penalty of the excluded volume effect. This model underscores the inherent lack of cohesion among FG-Nups, resulting in their mutual repulsion. This repulsion can be attributed, for instance, to electrostatic interactions occurring between the positively charged segments of FG-Nups (Peyro et al., 2021). This perspective diverges from that of alternative models scrutinized in the subsequent sections of this review, which accentuate the cohesive tendencies of FG-Nups arising from hydrophobic interactions between them.

### 2.3 Oily spaghetti model

The term “oily spaghetti” was first published by Ian Macara in 2001 and was used to describe the way that FG-Nups could potentially be arranged within the NPC (Macara, 2001). In the review article, Macara provided an overview of the molecular basis for nuclear transport, in particular the behaviors of NTRs and how they interact with NESs and NLSs to facilitate large cargo passage through the pore. Macara proposed that the FG-Nups form a sieve-like structure dominated by hydrophobic interactions that cargo carrier molecules can dissolve into, thus permitting the facilitated transport of cargo (Weis, 2007) (Figure 2B). This is an early example of a model that focuses on the hydrophobic interactions among FG-Nups that could allow for the formation of a cohesive, yet mobile structure within the pore, as opposed to the more repulsion-focused model of the polymer brush. Assuming the central channel of the pore which allows for the passive diffusion of small particles is approximately 10 nm in diameter, Macara approximated that the FG-Nups could form a spaghetti-like structure that lines the inside of the pore in a 7-nm-thick lining. It is important to note that this model does not present a clear explanation as to why the central channel would be left open for passive diffusion and, therefore, leaves the door open for a few possibilities. Perhaps molecules that are small enough could passively diffuse between the “spaghetti,” or the FG-Nups could reach into the center and block passive diffusion or force it to redirect from the center to occur more peripherally. From the perspective of Gibbs free energy, the idea behind this model is that the ∆G required for conformational changes in the FG-Nups is low enough that they can move freely; however, due to transient interactions between the FG repeats, the “spaghetti” somewhat clumps together onto the inner wall. The balancing act between random motion and transient interaction is the impetus for facilitating transport through the pore because the cargo–carrier proteins will also transiently interact with the FG-Nups. Because of the random movement assumed by this model, the FG-Nups themselves do nothing to impart directionality to the transport of particles. Directionality is instead attributed to the assembling and disassembling of the cargo–carrier complexes by RanGTP, which produces a ratcheting mechanism where cargo becomes trapped in its destination after its carrier has been removed. This ratcheting mechanism has been displayed by simulation as well as *in vivo* using single-molecule fluorescence microscopy (Lowe et al., 2010; Mincer and Simon, 2011).

Utilizing super-resolution single-point edge-excitation sub-diffraction (SPEED) microscopy, a study focusing on single-molecule dynamics has unveiled compelling findings (Ma et al., 2012). These findings revealed discrete pathways undertaken by individual particles during passive and facilitated transport through the NPC. The small fluorescently tagged molecules (<40 kDa) including single dyes, dyes bound to dextran, insulin, α-lactalbumin, and GFP showed a very high density in the center of the pore. The smallest particle, single fluorescein dyes, occupied the largest diameter region of approximately 37 nm, while the largest sub-40 kDa construct of Alexa Fluor-tagged GFP occupied approximately 17 nm, and the candidates in between generally occupied a region with decreased diameter with increased molecular mass. The transit of a 102-kDa construct of impβ1 bound to four Alexa Fluor dyes, and a 217-kDa construct of two GFPs, an NLS, and four Alexa Fluor dyes were tracked to measure the spatial densities of TRs and large molecules under facilitated transport, respectively. It was found that in both cases, the particles largely remained peripheral to the center of the pore. The tagged cargo-free Impβ1 was absent in a 23-nm-diameter region in the center, and the complexes of cargo/Impβ1 left an 8-nm-diameter hole and had a higher density closer to the inner surface of the NPC scaffold than those of Impβ1 alone. Discerning the differentiation between passive diffusion zones within the pore’s core and facilitated diffusion along its periphery might align with the discrete pathways proposed by the oily spaghetti model.

Additionally, within the context of the oily spaghetti model, FG-Nups are envisioned as being randomly scattered, forming transient connections without a defined pattern. Subsequent models have expanded on this notion, introducing a more organized representation of hydrophobic interactions.

### 2.4 Hydrogel model

The hydrogel model of the NPC, also known as the selective phase model, is based primarily on hydrophobic interactions of both FG-Nups with themselves and with cargo passing through the NPC (Figure 2C). This model was first described by Ribbeck and Görlich (2001). In this paper, they investigated the properties of facilitated translocation through the NPC under the selective phase model name, which they later expanded upon and described in 2007 as the hydrogel model (Frey and Görlich, 2007). The general concept of this model is that FG-Nups combine to form a homogenous meshwork, where the hydrophobic FG repeats interact with each other, forming a series of small gaps between them. These gaps allow for small particles to passively diffuse through the NPC, while larger particles must interact with the hydrophobic regions to displace the FG-Nups and facilitate their transport through the pore (Frey et al., 2006; Mohr et al., 2009). The paradox present is that this large cargo that needs to interact with the hydrophobic regions must efficiently pass through the pores while also having sufficient interactions with the FG-Nups to break through the hydrogel barrier. The many hydrophobic interactions between both the FG-Nups and the cargo passing through must therefore be sufficiently strong, as well as weak enough to dissociate and allow for passage. The efficient transport of large cargo is believed to be assisted by the re-association of FG-Nups behind the transiting cargo as it is displaced through the pore.

The hydrogel-forming properties of the yeast FG-Nup known as Nsp1 were investigated both *in vitro* and *in vivo* by Frey et al. (2006). An aqueous solution with a millimolar concentration of wild-type FG-repeat domains from Nsp1 was placed in a silicon tube and naturally formed into a transparent gel that could remain stable up to 95℃ . The gels could, however, be dissolved using the chaotropic agent guanidinium chloride, which supports the primary cohesive force being a noncovalent interaction between polypeptide chains. Samples of these FG-repeat domains were mutated, with each of the 55 phenylalanine amino acids being replaced by serine, hence removing the hydrophobic contributors to the repeats and replacing them with polar, hydrophilic contributors. These mutated regions, when treated in the same manner as the gel-forming wild-type counterparts, remained dissolved in the solution even at concentrations ∼four times greater than that of the wild-type, which shows that the hydrogel properties of FG-Nups are made possible by interactions with hydrophobic phenylalanine. To test the same mutations *in vivo*, yeast with their copies of Nsp1 were deleted. The yeast could recover from this lethal mutation if treated with replacement by Nsp1, even if their FG repeats were removed. The mutated version of the protein with the serine substitutions, however, could not rescue the yeast, which shows that even the removal of FG-repeats can be tolerated *in vivo*, but conversion to more hydrophilic structures cannot be tolerated, displaying the potential cruciality of the hydrophobic meshwork formation. Interestingly, a separate mutation that replaced the phenylalanine with tyrosine residues was still able to form a gel *in vitro*, albeit a less homologous one, suggesting that hydrophobic interactions alongside potentially stacking interactions could be factors contributing to the behaviors of FG-Nups.

It has also been found that the FG domains of at least 10 *Xenopus* Nups are capable of forming hydrogels and that Nup98 creates a stricter sieve than other tested Nups (Labokha et al., 2013). Recently, the hydrogel model has been the basis for a study on the effects of dipeptide repeats (DPRs) in the NPC and found that a synthetic hydrogel mimicking FG interactions was disrupted by arginine-rich DPRs, preventing the entry of Impβ (Friedman et al., 2022). These DRPs are associated with *C9orf72* repeat expansions, which have been found to be the leading cause of frontotemporal dementia and amyotrophic lateral sclerosis (Smeyers et al., 2021). Both the hydrogel model and the oily spaghetti model imply that the behavior of FG-Nups is primarily influenced by hydrophobic interactions. However, the hydrogel model inherently lacks distinct pathways for passive or facilitated diffusion, given that passive diffusion could potentially occur through any sufficiently large gaps within the FG-Nup structure. Moreover, it is essential to highlight that the hydrogel structure of FG-Nups has been exclusively synthesized under controlled laboratory conditions (*in vitro*) and has not been observed within a natural NPC environment.

### 2.5 Reduction of dimensionality model

The reduction of dimensionality (ROD) model was proposed by Peters (2005). This model suggests the presence of a dense layer of FG domains that coats the inner wall of the NPC as a continuation of the FG motifs of the cytoplasmic fibrils. Furthermore, the TRs required for facilitating transport are proposed to act as ferries that bind to cargo and stick to the dense layer of FG domains (Figure 3B). The name of this model refers to the idea of reducing the dimensions of transport from 3D to 2D, explaining the movement of TRs as a 2D random walk, through which they randomly traverse this dense FG surface until reaching their exit. The principle of ROD is a method of increasing the rate of reaction, i.e., between an enzyme and ligand. A 2D surface on which a ligand can walk across provides an increased likelihood of it locating and successfully interacting with its enzyme, given that the enzyme is hosted on the same surface. Conceptually, transport complexes in the NPC can be thought of as ligands, and the nuclear or cytoplasmic exit from the pore is the target enzyme (Adam and Delbrück, 1968). The ROD model portrays the structures and actions of the FG-Nups by proposing that they coat the inner surfaces of the pore, establishing a foundation for the erratic mobility of TRs. Additionally, the model suggests the creation of a narrow central pathway within the pore for transpiration of passive diffusion. As per this model, passive diffusion is facilitated through an 8–10-nm-diameter space at the core of the pore, encircled by a loosely interconnected network of hydrophilic polypeptide chains. This network functions as a selectivity barrier, impeding the passive diffusion of larger molecules. Different from previous models, the ROD model does provide a possible explanation as to how passive and facilitated diffusion would be spatially distinct based on the proposed collapse of the FG-Nups. However, it does not fully explain why the inner diameter that permits passive diffusion would have a size of 8–10 nm and not larger as a result of the FG-Nups further retreating out to the periphery.

While the ROD model was proposed as a hypothetical model, a study performed by Schleicher et al. evaluated the potential for the 2D walk behavior of traversing particles predicted by the model *in vitro* using optical trapping and single-particle tracking (Schleicher et al., 2014). The experimental design consisted of a layer of surface-tethered Nup153 being moved toward an optically trapped colloidal probe coated with Impβ and using a photonic force microscope to measure the probe’s movement with nanometer-microsecond spatiotemporal resolution. The experiment was performed with different concentrations of impβ solutions, ranging from 0.5 to 30 µM. It was found that the “jump-to-contact” forces gradually decreased as the environmental concentration of impβ increased, along with an increase in 2D mobility of the probe. At 30 μM, which is a physiologically reasonable concentration of importins (Paradise et al., 2007), the probe was shown to move across the X-Y plane nearly unhindered in a 2D walk while remaining bound. The assumed causes of these effects, and the general effect of TRs in the pore, are explored further in the next section of the paper. Ultimately, this study has shown that under certain cellular conditions, the ROD model could accurately predict the type of movement imparted upon receptor-dependent cargo in the NPC.

### 2.6 Forest model

In 2010, Yamada et al. published a study performed on purified *Saccharomyces cerevisiae* Nups that described the heterogeneous nature of FG-Nups, specifically proposing that there are two distinct types of FG-Nups, each with different chemical properties and spatial arrangements reminiscent of a “forest” (Yamada et al., 2010). Some FG-Nups appear in the form of a collapsed coil with low charge content near the NPC anchor domain, while others contain extended coil regions of higher charge content with collapsed coil or folded globule on the end opposite to the anchor domain (Figure 3D). The first set of FG-Nups, with collapsed coils adjacent to the anchor domain, are reminiscent of “shrubs,” whereas those that possess extended coils appear as “trees,” hence the name “forest model.” Because of their charged, extended coil regions, the “trees” possess more flexibility than the “shrubs,” which are more constrained to the inner surface of the pore. This model represents a synthesis of some of the properties described in previous models. While the polymer brush emphasizes electrostatic repulsion and non-cohesiveness and other models such as the hydrogel model emphasize cohesive hydrophobic interactions, the forest model follows both models to produce a more complete picture of the NPC.

In general, the “shrubs” tended to be less than ∼10 nm in hydrodynamic diameter, while the “trees” were found to be around double that, with their collapsed coil regions making up a little more or less than half of that value depending on the particular Nup. It was determined that the best way to predict whether an FG-Nup is a “shrub” or “tree” is by the ratio of the charge to hydrophobic amino acids. Nearly all the collapsed coil FG-Nups analyzed had a charged/hydrophobic amino acid ratio of less than 0.2, whereas all FG-Nups classified as relaxed or extended had ratios ranging from 0.7 to 1.4. One of the most important aspects of this model is the spatial separation of the largely hydrophobic FG regions and the heavily charged regions. Because the collapsed coil regions tend to aggregate with each other as a result of the hydrophobic effect, and because the “shrubs” and “trees” have their collapsed regions near the periphery and near the center of the pore, respectively, a distinct new region is formed that has not been described by other models. This new region appears around the radial midpoint from the center of the pore and corresponds to the extended, highly charged regions created by the “trees.” This model, thus, proposes two distinct transport zones, one through the very center of the pore and the other in this newly described region, termed by Yamada et al. as “zone 1” and “zone 2,” respectively. It is important to note that although this was the first study to mention the forest model by name, it was not the first to suggest that the NPC possessed multiple gates formed by different properties of FG-Nups. Patel et al. (2007) found evidence to support a two-gate model of the NPC. One of the gates they proposed was formed by hydrophobic attractions that formed a selective phase barrier in the center of the pore, as described by the hydrogel model, while another region contains a non-cohesive virtual gate more peripherally localized (Patel et al., 2007). This description seems to point to the same behaviors of FG-Nups as described by the forest model.

Liashkovich et al.(2012) used AFM to investigate the binding patterns of two different particles in *X. laevis* oocyte NPCs to resolve whether or not the forest model may be accurate (Liashkovich et al., 2012). The first particle was wheat germ agglutinin (WGA), which interacts with N-acetylglucosamine-modified (GlcNAc) Nups such as Nup62, and the other was the mutated TR Impβ^45–462^, which specifically binds to FG regions. While the WGA was found near the periphery of the pore, the Impβ^45–462^ was found primarily in the center of the pore rather than the periphery, suggesting the presence of the “trees” as described by this model. In addition, this study found that despite the WGA binding primarily to the periphery of the pore, it provided a stronger barrier for passive diffusion than the Impβ^45–462^, suggesting the presence of the alternate transport route described as “zone 2” in the forest model.

Eibauer et al. (2015) paired cryo-ET with sub-tomogram averaging on the NPC scaffold of the *X. laevis* oocyte and showed a structure with the proposed central and peripheral transport routes as described by the forest model (Eibauer et al., 2015). In addition, coarse-grained simulation work studying the NPCs of *S. cerevisiae* found that longer FG-Nups can simultaneously form a dense FG domain and charged disordered region, described as a diblock polymer reminiscent of “trees” (Ando et al., 2014). It was then found that additional low-charge, FG-rich single-block Nups in the simulation could conform into a “shrub” shape and help stabilize a more open conformation in the presence of large cargo in the pore’s center (Ando and Gopinathan, 2017).

### 2.7 Gradient model

The gradient model, also known as the affinity gradient model, was proposed by Ben-Efraim and Gerace (2001). According to this model, different Nups are localized at different positions within the NPC, forming an affinity gradient for TRs and cargo along the transport pathway. As cargo passes through the NPC, it encounters these different Nups with increasing binding affinity, which is the impetus for directional transport (Figure 3A). Other models have assumed that the FG-Nups impart no directionality on transport, but that directionality is created by concentration gradients between the nucleoplasm and cytoplasm and the RanGTP cycle. The gradient model represents a bold departure from this assumption, proposing that FG-Nups play a role in directionality beyond random, transient interactions. While it does seem to be generally accepted that an affinity gradient is not the driving force of directionality, there may be particular cases in which it does play a role, as is explored later in this section.

In their study, the researchers measured the affinity of import complexes for Nups that interact with Impβ, namely, Nup358, the Nup62 complex, and Nup153. These Nups were selected based on their localization within the NPC, representing early, intermediate, and late binding sites, respectively. The movement of Impβ through the NPC involves sequential transfer from Nup358 to the Nup62 complex and then to Nup153. This directional bias is attributed to the increasing affinity of the transport complex for the Nup binding sites encountered sequentially; although the transfer between Nups can occur in both forward and backward directions, the increasing affinity for more distal Nups promotes unidirectional transport (Ben-Efraim and Gerace, 2001). The release of the cargo complex from the terminal Nup binding site is mediated by RanGTP (Görlich et al., 1996).

It has been proposed that the transfer of transport complexes between two Nups may involve a cooperative reaction, where an input cargo complex bound to one Nup is induced to release from its binding site upon interaction with a second Nup (Ben-Efraim and Gerace, 2001). Researchers have found that different regions of Nup358 and different subunits of the Nup62 complex bind to Impβ with similar affinity. However, they were unable to provide experimental data to support this theory due to the inability to measure the individual interactions between Nup153, Nup62, Nup58, and Nup358 fragments.

Azimi et al. designed an agent-based modeling (ABM) simulation to analyze the interactions between Impβ and FG-Nups and tested whether or not the affinity gradient could significantly improve the transport rate (Azimi and Mofrad, 2013). An ABM is essentially a method of simulating interactions between individual objects known as “agents,” and it has been used in a wide variety of applications (Macal and North, 2009). This simulation is based on a simplification that interactions between Impβ and FG-Nups occur with a single probability based on bulk affinity values, and because it is assumed that this binding occurs rapidly and often enough, this simplification is valid. One of the findings of this paper is that in the models with no affinity gradient, the transport rate for Impβ peaks at an affinity of approximately 86.24 µM and decreases at higher or lower affinities. It was also found that transport rates of Impβ are most impacted by the affinity of the nuclear basket Nups, less so by that of central channel Nups, and negligibly by the affinities of cytoplasmic Nups. Ultimately, it was determined that import efficiency is more dependent on the concentration gradient of RanGTP, highlighted by the fact that even a complete reversal of the affinity gradient does not prevent import. However, the affinity gradient does marginally increase import efficiency and, therefore, could still be a regulatory mechanism for the NPC.

A recent paper by Shen et al. presents synthetic, programmable recreations of the NPC, designed by using DNA corrals that can be associated with different Nups (Shen et al., 2023). The study was performed to analyze the nuclear import behaviors of human immunodeficiency virus 1 (HIV-1), specifically the interactions between the capsid and various Nups, because it has recently been shown that these large capsids can pass through the pore intact (Zila et al., 2021). It was found that multiple copies of Nup153 or Nup358 are required for successful import and that the affinity between the capsid and Nup153 is greater than that of Nup358. It was also found that Nup62 tends to self-interact, creating a barrier for the capsid. Even when 32 copies of Nup62 were grafted to the outside of a 60-nm channel, they still managed to come together at the center of the pore. This paper proposes a three-step mechanism of capsid penetration involving an affinity gradient. The lower-affinity interactions between the capsid and Nup358 at the cytoplasmic region allow for docking, and then interactions with the stronger-affinity Nup153 in the nuclear basket of the pore orient the cone-shaped capsid lengthwise, overcoming the barrier provided by Nup62 in the central channel and pulling it inside the NPC. Vial et al. utilized a combination of SMLM and AFM to show that the nuclear basket is flexible enough to fold inward toward the center of the pore (Vial et al., 2023). This could help explain how Nup153 can cling on to the capsid and assist in its import. It may be the case that, even if the gradient model is not the driving force for smaller particles, it plays a critical role in transporting larger particles, especially if they require a particular orientation for successful transport.

### 2.8 Dilation model

The term “dilation model” is introduced here not to supplant existing models but to complement them, enriching our understanding of the NPC as a whole. It is firmly established that NPCs exhibit the ability to expand and contract (Feldherr and Akin, 1990; Kiseleva et al., 1998; Shulga and Goldfarb, 2003; Beck et al., 2004). However, the exact mechanisms behind this behavior and its implications for transport remain subjects of ongoing investigation. Despite this well-established pore behavior, the other models do not incorporate it into their explanations of NPC functionality. Thus, the dilation model fills an essential gap in the puzzle. In a sense, this model extends beyond the others previously described. Rather than concentrating solely on cargo–Nup interactions within the pore, the dilation model focuses on how external environmental factors induce conformational shifts in the NPC (Oberleithner et al., 2000; Mooren et al., 2004; Shahin et al., 2005; Kastrup et al., 2006; Lim et al., 2006; Zimmerli et al., 2021). Notably, the measured diameter of the NPC can even be altered by the techniques used for pore analysis, and purified NPCs often exhibit smaller diameters than those measured *in situ* (Schuller et al., 2021; Akey et al., 2022).

Calcium has been shown to play a role in regulating the size and shape of the NPC (Moore-Nichols et al., 2002; Erickson et al., 2006); however, it is not clear whether or not this action has a significant impact on transport through the pore (Sarma and Yang, 2011). Cho et al., investigating the regulation of the nucleocytoplasmic transport of truncated ataxin-3 (ATXN3) proteins in *Drosophila* neurons, found that decreasing intracellular calcium levels can reduce the nuclear accumulation of ATXN3; however, this is likely a result of calcium-mediated regulation of CBP rather than the constriction of the pore (Cho et al., 2022).

In general, it has been observed primarily through AFM and SEM in *X. laevis* oocytes that in the presence of calcium, the NPC has a wider diameter on either the cytoplasmic side (Jäggi et al., 2003), nuclear side (Stoffler et al., 1999), both (Erickson et al., 2006), and/or displaced central mass (Wang and Clapham, 1999; Moore-Nichols et al., 2002; Mooren et al., 2004). Calcium stores are located within the NE and can be released via inositol triphosphate (IP_3_) receptors, and pore conformation can be regulated by IP_3_ and other effectors of the receptor such as the agonist adenophostin A or inhibitor xestospongin C (Moore-Nichols et al., 2002; Erickson et al., 2006). For a more in-depth review of the effects of calcium on the NPC, see Sarma and Yang (2011). It has also been shown via AFM that the introduction of 1 mM of ATP has a transient contracting effect on the shape of the NPC, causing the height of the pores to increase alongside a decrease in the diameter (Rakowska et al., 1998). Another molecular effector of NPC shape is CO_2_, and it has been shown by AFM that exposure to 5% CO_2_ led to significant closure of the pore within minutes, an effect that was slowly reversible (Oberleithner et al., 2000).

Shahin et al. discovered that the steroid dexamethasone (dex), when injected into *X. laevis* oocytes, causes a two-step mechanism involving the dilation of the NPC (Shahin et al., 2005). In the first step, dex-initiated proteins (DIPs) bind to the NE, which causes dilation of NPCs from approximately 82–110 nm, and then, the DIPs enter the dilated pores. To explore the limits of this effect, the same laboratory reproduced the procedure, this time measuring the effect of dex exposure after longer periods of time (Kastrup et al., 2006). After a period of 5–11 min, dilated pores reached diameters of approximately 140 nm. Even more interestingly, this treatment produced a subpopulation of so-called “giant pores,” some of which reached diameters of approximately 300 nm. Using nuclear envelope electrical conductivity (NEEC) to measure the permeability of small ions through the NPC, it was found that the dex-injected cells had an increased in the NEEC by 125% compared to non-treated and control cells and that the treated cells returned to baseline levels after around half an hour. This extreme dilation behavior could lead to potential advancements in gene therapies because entrance into the nucleus is one of the major barriers to introducing genes into eukaryotic genomes. For example, researchers have attempted to use adeno-associated viruses as a way of passing genes into the nucleus because the large virus capsid can pass fully intact through the NPC (Kotin et al., 1990; Deyle and Russell, 2009); however, the transport efficiency through the pore has been found to be quite low (Kelich et al., 2015). Therefore, treatments that could dilate the pore may be useful for increasing the import efficiency of these capsids. It also could prove useful for treating diseases that have been shown to hinder pre-ribosomal subunit export such as Diamond Blackfan anemia (Léger-Silvestre et al., 2005; Choesmel et al., 2007). Because the pre-40s and pre-60s ribosomal subunits are among the largest cargo to export through the pore, a significant increase in diameter could increase their export efficiency (Zemp and Kutay, 2007; Gerhardy et al., 2014).

Zimmerli et al. utilized cryo-EM to examine the mechanosensitive properties of the NPC relating to cellular stress and nuclear volume (Zimmerli et al., 2021). Under conditions of hyperosmotic shock and cellular energy depletion, the nuclear volume is decreased, which results in loss of NE tension, causing the central channel diameter of the NPC to contract from approximately 70 nm to less than 50 nm, in both cases reducing passive transport as measured by freely diffusing GFP. This study was performed using *Schizosaccharomyces pombe* cells, and energy depletion was induced via exposure to nonhydrolyzable 2-deoxy-glucose and antimycin A, while osmotic stress was provided by sorbitol treatment. Another recent *in vivo* study by Pulupa et al. utilized polarized total internal reflection fluorescence microscopy (pol-TIRFM) to study conformational changes in individual NPCs within HeLa and Hap1 cells (Pulupa et al., 2020). Their developed technique utilized the shallow illumination depth of TIRF microscopy to illuminate the bottom of the nucleus, exciting Nup–mEGFP fusion proteins with either parallel (p) or perpendicular (s) polarized light relative to the central axis of the NPC. By measuring the p:s ratio, they determined the conformational change in the pore indicated by orientational changes in the selected Nup fusion protein, which included Nup133, Nup93, Nup58, and Nup54. It was found that Nup54 and Nup58 had orientational shifts after cellular starvation, whereas Nup133 and Nup93 had no significant changes. It was also found that altering karyopherin content in the pore changes the orientation of Nup54, which may indicate that changes in the transport state of the NPC induce changes in the geometry of the scaffold.

**FIGURE 4:**
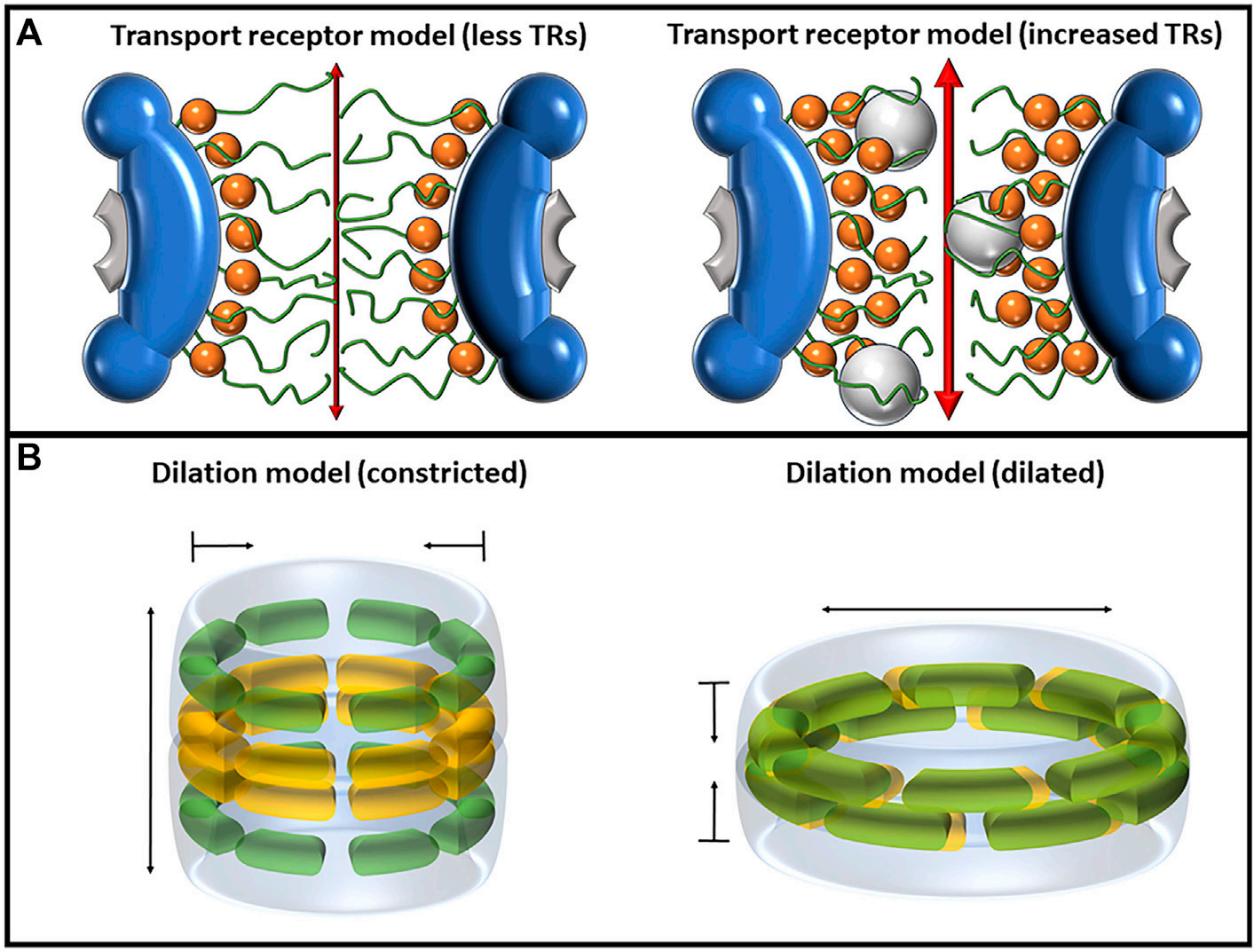
Depictions of the transport receptor and dilation models, each of which has mechanisms of altering the diameter of the central pore. **(A)** The transport receptor model as shown with a single layer of TRs (left) and two layers of TRs (right). The binding of TRs, depicted as orange spheres, to FG-Nups pulls the Nups away from the center of the pore, increasing both passive and facilitated transport rates. **(B)** The dilation model as predicted by Melčák et al. showing the constricted state (left) and dilated state (right). Nup58 is shown in yellow, and Nup54 is shown in green. In the dilated state, the two Nups interact with each other, forming eight Nup54–Nup58 dodecamers in the midplane that can slide against each other to modulate the diameter of the pore.

The exact molecular mechanisms of pore dilation and contraction are still under investigation; however, research by Melčák et al. has been unfolding the potential mechanisms for such behavior (Melcák et al., 2007; Solmaz et al., 2013; Sharma et al., 2015). Melčák et al. (2007) proposed a mechanism for NPC dilation based on circumferential sliding between two Nup58/45 dimers (Melcák et al., 2007). Each Nup58/45 protomer is folded into a hairpin structure with N and C α helices separated by a short loop; hydrophobic interactions between two protomers form a dimer, and tetramerization occurs via a continuous electrostatic surface with alternating positively and negatively charged amino acids between adjacent, parallel N-helices on opposite dimers. This electrostatic interface could allow for sliding along the helical axes approximately 1.1 nm and, given the eight-fold symmetric arrangement of these tetramers, could permit a channel diameter increase by approximately 3 nm. It was in their 2013 paper that this group introduced the “ring cycle” model, which was later expanded upon in 2015, and is used as an explanation to further explain how pore dilation could occur (Solmaz et al., 2013; Sharma et al., 2015). The central mechanism behind this model lies within interactions between Nup54 and Nup58. In the constricted state, it is believed that Nup58 forms an eight-fold homotetrameric ring around the midplane of the NPC, while Nup54 forms two similar rings above and below the midplane. In the dilated state, the two Nups interact with each other, forming eight Nup54–Nup58 dodecamers in the midplane that can slide against each other to modulate the diameter of the pore. The center of the Nup54 homotetramers contains highly conserved polar residues that can also interact with polar residues along Nup58, both of which provide a level of intermolecular instability that could permit low-energy interconversion between their homo-oligomeric and hetero-oligomeric states. Hence, the “ring cycle” name refers to the process of interconversion between the large Nup58–Nup54 and the three smaller rings of homotetramers (Figure 4B).

### 2.9 Transport receptor model

The transport receptor model aims to elucidate the influence of TRs on both the structure and behavior of FG-Nups. It is worth noting that the term “transport receptor model” is introduced here, although it primarily represents a synthesis of observations concerning TR behaviors within the NPC. The presence of TRs within the NPC brings about alterations in the architecture of FG-Nups, triggering conformational changes that can impact nucleocytoplasmic transport (Aramburu and Lemke, 2017; Tan et al., 2018). Heightening the concentrations of the TR Impβ has been demonstrated to not only enhance the efficiency of transporting signal-dependent and -independent cargo but can also substantially accelerate transit speeds by at least 10-fold (Yang and Musser, 2006) (Figure 4A).

One group investigating the mechanisms for this behavior is Lim et al., who have greatly advanced the current understanding of the effects of TRs in the NPC across the last two decades (Lim et al., 2006; Lim et al., 2007; Schoch et al., 2012; Kapinos et al., 2014; Wagner et al., 2015; Barbato et al., 2020; Kalita et al., 2022). In a 2006 study investigating the *in vitro* dynamics, it was demonstrated through AFM analysis of tethered Nup153 that treatment with a less polar solvent (5% 1,2-hexanediol) collapses the FG-Nups, which can be reversed by replacement with PBS (Lim et al., 2006). This collapsing mechanism of Nup153 induced by an increase in hydrophobicity became a basis for the karyopherin-centered model of the NPC. By covalently tethering Nup153 to gold nanodots, Lim et al. effectively produced a simplified NPC-like environment where the effects of karyopherins could be measured by AFM (Lim et al., 2007). They found that like the hexanediol treatments, increasing the concentration of Impβ1 reversibly decreased the reach of the FG-Nups. Concentrations of Impβ1 in PBS from 0 M, 115 fM, 2.5 pM, and 33 nM corresponded to a respective decrease in Nup lengths of 29.1, 17.9, 13.7, and 11.3 nm. Because RanGTP is the mechanism by which impβ is dissociated from FG-Nups in the termination of nuclear import *in vivo*, it was speculated that treatment with RanGTP could be an effective mechanism for reversing the collapse of these Nups (Kalab et al., 2002). It was found that after treatment with 0.33 nM Impβ, increasing the concentrations of RanGTP from 0 M, 0.1, 0.2, and 0.56 nM corresponded to an increased Nup length of approximately 7.4, 10.4, 16.2, and 34.5 nm, respectively.

A further *in situ* investigation of Nup62 utilizing surface plasmon resonance (SPR) measurements found that there are multiple phases of interaction between Impβ and these FG-Nups (Schoch et al., 2012). Based on their results, they proposed that Impβ binds to FG-Nups, initially causing a slight collapse, but as more Impβ proteins bind tightly to this layer, the steric repulsion induces the recovery of the Nups, and they extend outward again. In addition, they proposed that after sufficient binding of Impβ, a second layer can stack on top of the first layer with weaker interactions than the first layer, causing a “pile-up” effect. These effects were further investigated using SPR, and it was found that Nup214, Nup62, and Nup153 are all capable of extending to accommodate several layers of Impβ. At physiological Impβ levels, this mechanism can produce a closely packed Impβ layer against the inner surface of the pore, and a looser and more fast-moving layer of Impβ that appears after the first layer is saturated (Kapinos et al., 2014). The proposed mechanism for this is that the first layer of Impβ has many interactions with the FG regions, and the next layer now has fewer potential interaction sites and, therefore, moves more freely. Furthermore, NFT2 was found to have faster transport kinetics than Impβ, which is believed to be facilitated by the occupancy of Impβ already fulfilling many of the potential FG interactions, which would otherwise make transit slower (Wagner et al., 2015).

High-speed super-resolution light microscopy has corroborated the observation that the presence of Impβ1 within the pore can induce a collapse of FG-Nups, thereby modifying both the facilitated and passive transport routes. Notably, at a concentration of 15 μM Impβ1, the permeability for 70-kDa dextran molecules increased by enlarging the central passive diffusion channel. These molecules, which typically struggle to diffuse through the NPC at lower Impβ1 concentrations, experienced enhanced permeation (Ma et al., 2012). Additionally, this methodology has recently enabled the three-dimensional tomography of the FG–Nup barrier, unveiling its interactions with multiple TRs in native NPCs (Ma et al., 2016). The findings suggest that each TR occupies a distinct interaction zone within the FG–Nup barrier. Notably, two key TRs, Impβ1 and exportin 1 (CRM1), surpass other TRs in their binding to FG-Nups. Furthermore, the TRs are capable of modifying the tomography of the FG–Nup barrier, exerting influence on each other’s pathways, particularly in situations characterized by intense competition.

Kalita et al., using SPR to examine the behaviors of Impβ and CRM1 in relation to interactions with FG-Nups, showed that these TRs are required to bolster the inner structure of the NPC and that leakage through the pore was a symptom of low TR levels (Kalita et al., 2022). Silencing of Impβ produced a 16% increase in the nuclear/cytoplasmic ratio of 2xEGFP-NES, which signifies that leakage increased NPC permeability. Further increased levels of Impβ silencing induced cell death. It was also discovered that CRM1 is capable of compensating for a loss of Impβ to still maintain structure in periods of low Impβ content. Both Impβ and CRM1 were found to bind comparably to FG-Nups, and a silencing of Impβ measured by an immunofluorescent signal decrease of approximately 12%–18% in the NE corresponded with an increase in the CRM1 signal of up to 121%, suggesting that CRM1 is being recruited to counteract the lack of Impβ in the pore. Ultimately, the findings contributing to the transport receptor model implicate TRs as being more important to the structure and function of the NPC than previously understood, pointing to TR–FG interactions potentially being comparably critical to FG–FG interactions.

## 3 Nuclear transport routes taken by transmembrane proteins

The nuclear envelope comprises both the outer and inner nuclear membranes (ONM and INM, respectively), establishing a boundary between the nucleus and the cytoplasm. While the models previously discussed primarily center around the targeted bidirectional movement of soluble molecules via NPCs embedded at the fusion site of the ONM and INM (Bondos et al., 2021), the route taken by nuclear envelope transmembrane (NET) proteins entering the nucleus remains a point of contention. Theoretically, the transport into the INM could manifest through either NPC-dependent or NPC-independent pathways (Mudumbi et al., 2020). Despite the observation of NPC-independent transport during viral egress (Kuersten et al., 2001; Weis, 2002), no study has furnished evidence of its application in the import of INM proteins.

**FIGURE 5:**
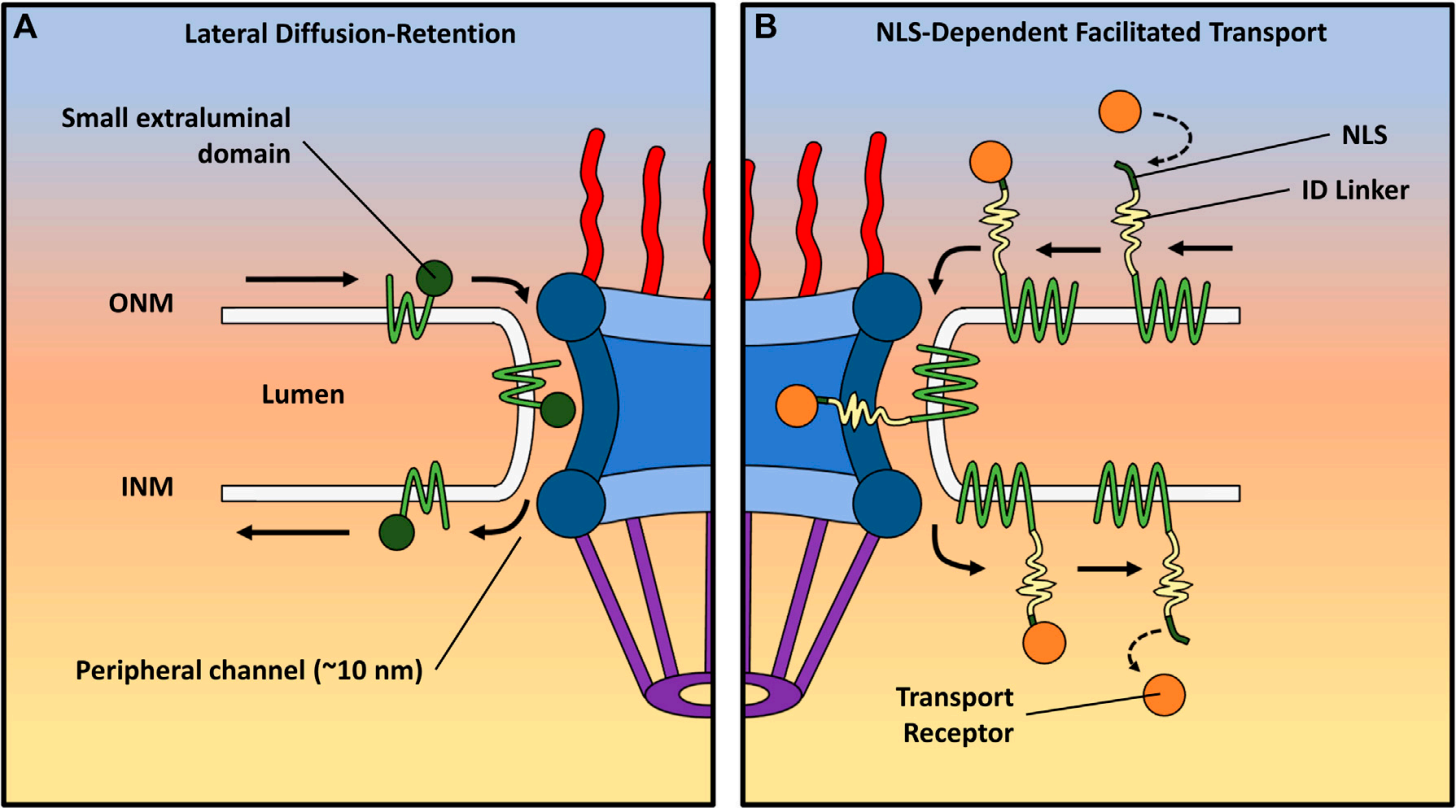
Depiction of two distinct transport mechanisms for transmembrane proteins. Each route displays the trafficking of NET proteins from the ONM to the INM. **(A)** The lateral diffusion–retention model proposes that INM proteins move freely between the ONM and INM, with their directionality imparted by binding partners within the INM. This model confines these proteins to peripheral NPC channels (∼10 nm wide), limiting the size of their extraluminal domains to approximately 60 kDa. **(B)** The nuclear localization signal (NLS)-dependent facilitated transport model suggests that extended regions of ID alongside NLSs play a crucial role in the import of NET proteins with larger extraluminal domains. The ID regions can slide through the scaffold of the NPC, inserting NLS-containing domains bound to TRs into the central channel. The TRs can then interact with FG-Nups in the pore in a manner resembling the facilitated transport of soluble proteins.

Instead, two prominent NPC-dependent models come into play: the free lateral diffusion–retention model and the nuclear localization signal (NLS)-dependent facilitated transport model (Figure 5). Both models stipulate that the transmembrane domain of INM proteins remains integrated within the nuclear envelope as they traverse from the ONM to INM. However, crucial disparities differentiate these mechanisms. In the lateral diffusion–retention model, transmembrane proteins diffuse freely within the membrane spanning the ONM and INM, and their directionality stems from retention by binding partners within the INM. This model confines INM proteins to multiple peripheral NPC channels, as indicated cryo-EM, with an approximate width of 10 nm (Talcott and Moore, 1999; Stoffler et al., 2003; Von Appen et al., 2015; Samudram et al., 2016; Baade and Kehlenbach, 2019). This width constrains the nucleoplasmic domains of INM proteins to approximately 60 kDa, assuming a globular structure, resulting in a hydrodynamic radius of approximately 10 nm. A more linear configuration could potentially allow these proteins to navigate these channels with a smaller radius, in line with the transport direction. This 60-kDa limitation has been experimentally confirmed (Wang and Brattain, 2007; Tai et al., 2023) and aligns with the diverse NETs identified in the nuclear envelope (Kirli et al., 2015; Popken et al., 2015). However, most NETs are likely to encompass regions of intrinsic disorder (ID) within their nucleoplasmic domains rather than being rigidly folded. Several signals, including nuclear localization signals and ID regions in the nucleoplasmic domain of NET proteins, have been recognized as crucial for transport. Notably, certain transport receptors like importin alpha and beta, crucial for central channel transport (Hough et al., 2015; Milles et al., 2015; Best, 2017; Schuller et al., 2021), are too large to fit within the peripheral NPC channels. Nevertheless, these receptors have been demonstrated to facilitate NET transport in yeast (Görlich and Kutay, 1999; Ashkenazy-Titelman et al., 2020). Therefore, the NLS-dependent mechanism proposes that the presence of extended ID regions alongside NLSs in the nucleoplasmic domains of INM proteins is vital. These ID domains could theoretically extend through the core structure of the NPC, enabling the NLS-containing nucleoplasmic domain to reach the central channel (∼50 nm wide at its narrowest point). This arrangement would allow the NLSs to bind transport receptors and FG Nups, thereby resembling the transport of soluble proteins. RanGTP-dependence, potential NLSs, and TR associations are all traits that different NETs in both yeast (King et al., 2006; Liu et al., 2010; Gardner et al., 2011) and human cells (Ma et al., 2007; Turgay et al., 2010) have been observed to exhibit, which are characteristic of facilitated transport through the NPC.

Recent utilization of several advanced single-molecule techniques, including single-molecule fluorescence recovery after photobleaching (smFRAP), single-molecule Förster resonance energy transfer (smFRET), and high-speed virtual 3D SPEED microscopy, has facilitated the delineation of routes taken by various transmembrane proteins (Mudumbi et al., 2016; Mudumbi et al., 2020). Significantly, the findings have demonstrated that all NETs remain embedded within the membrane throughout their transport, offering functional insights into the mechanism of transmembrane protein movement. Importantly, only an estimated 9% of the numerous INM NETs, considering the combined presence of NLS and ID domains of appropriate length, are likely to possess the potential to engage transport receptors. These INM proteins exhibit distinct domains within the central and peripheral channels during transport, facilitating access to transport receptors for the domain in the central channel. Nonetheless, when central channel transport is impeded, these INM NETs can exclusively resort to utilizing peripheral channels for their passage. For example, the NET known as the lamin-B receptor (LBR) has both an ID region and a predicted NLS near its extraluminal N-terminus. Mudumbi et al. showed that a point mutation designed to inactivate the predicted NLS in the LBR caused a spatial density shift during the import of the N-terminus from the inside walls of the NPC to the peripheral channel (Mudumbi et al., 2020). They performed another experiment with the NET lamina-associated polypeptide 2-beta (Lap2β), deactivating a predicted NLS, and found a shift of the N-terminus from approximately 24 nm radially from the central axis of the pore to approximately 41 nm, where the peripheral channel is located. In addition to inactivating predicted NLSs in those NETs, the removal of the ID linker present in the N-terminal end of the LBR also showed a shift to the peripheral channel, suggesting that both NLSs and ID regions are required for the central channel transport of NETs. It is important to note that blocking the peripheral channels can lead to the inhibition of translocation through both channels. This underscores the role of peripheral channel transport as the default mechanism, which evolution has adapted to encompass aspects of receptor-mediated central channel transport, thereby ensuring the precise trafficking of specific membrane proteins. Interestingly, an INM protein involved in the LINC complex known as Sad1 and UNC84 Domain Containing 2 (SUN2) has been reported to have a functional NLS; however, neither mutations to that NLS (Turgay et al., 2010) nor Ran depletion prevents its successful import (Zuleger et al., 2011), and the two co-existing routes for NETs could help explain this observation.

Further research is necessary to comprehensively characterize the transport pathways of NETs, with the aim of achieving a more profound understanding. Furthermore, a deeper insight is required into the implications of various ID regions and NLS-free signals on the import pathway. For instance, a significant gap in knowledge still exists regarding the transport mechanism in cases of non-NLS signal sequence-mediated transport (Saksena et al., 2006). In this mode of transport, the precise manner by which an INM sorting motif is recognized by importin-α 16, followed by subsequent translocation through the peripheral channel of the NPC, remains unclear. Alternatively, an additional facilitative process could potentially be attributed to FG repeats on INM proteins (Kerr and Schirmer, 2011), which may interact with FG-Nups situated within the peripheral channel, thereby contributing to the overall transport mechanism.

## 4 Perspective

The NPC serves as a critical cellular structure responsible for governing molecular traffic between the nucleus and cytoplasm. Despite extensive investigations, deciphering the precise structure and behavior of the NPC remains a challenge, which is evident from the prevailing lack of consensus and the paucity of new models in recent years. This review delves into diverse viewpoints that illuminate the intricacies of the NPC, with the intent of unraveling insights into its underlying structure and functionality.

One crucial consideration pertains to the heterogeneous nature of the NPC. Various models have underscored distinct structural attributes and mechanisms, culminating in a range of proposed interpretations (refer to Table 1). Certain models center around the pivotal role of FG-Nups in constituting a selective barrier within the central channel, while others accentuate the interplays between NTRs and FG-Nups for cargo transportation. These divergences underscore the necessity of amalgamating multiple facets into a unified framework to holistically depict the NPC’s behavior. Indeed, the distinctiveness characterizing each model in this discourse makes it challenging to unequivocally endorse any single model’s accuracy.

**TABLE 1:** Overview of various models concerning the NPC, including the year of proposal, if applicable, and a comparative analysis of distinct characteristics. The abbreviation “n/c” denotes “not clear.”

Model and year proposed	Single or multiple channels for passive diffusion	Distinct passive and facilitated pathways	How do FG-Nups interact to form a selective barrier?	Does the scaffold change conformation?
**Plug** 1982	Multiple	n/c	n/c	n/c
**Gradient** 2001	n/c	Yes	Affinity gradient	n/c
**Oily spaghetti** 2001	Single	Yes	Hydrophobic	n/c
**Selective phase/hydrogel** 2001	Multiple	No	Hydrophobic	n/c
**Polymer brush** 2003	Single	Yes	Electrostatic	n/c
**ROD** 2005	Single	Yes	Hydrophobic	n/c
**Forest** 2010	Multiple	Yes	Hydrophobic/electrostatic	n/c
**Dilation** n/c	n/c	n/c	n/c	Yes
**Transport receptor** n/c	Single	Yes	Hydrophobic	n/c

As evident in this paper, each model rests on elements substantiated by empirical evidence. In striving to ascertain the most plausible model, the evaluation must weigh the robustness of differing data types. The authors are inclined to consider live cell and dynamic data as the most adept at describing the native NPC’s structure and function. Nonetheless, upon comparison (see Table 2), it becomes apparent that several disparate models draw validation from live cell and dynamic data, indicating that components of each model are accurate within specific contexts. This suggests the potential necessity for a fresh model that amalgamates elements from these diverse models to more comprehensively capture the intricacies of the NPC.

**TABLE 2:** Evidence supporting NPC models: summary of live cell and dynamic data availability, along with relevant papers.

Model	Live cell data?	Dynamic data?	Supporting evidence
**Plug**	No	Yes	**Cryo-EM** (Unwin and Milligan, 1982)
			**Cryo-ET** (Beck et al., 2004)
			**HS-AFM** (Sakiyama et al., 2016)
**Gradient**	No	No	**Biochemical assays** (Ben-Efraim and Gerace, 2001)
			**Simulation** (Azimi and Mofrad, 2013)
			**Synthetic NPC** (Shen et al., 2023)
**Oily spaghetti**	Yes	Yes	**Single-molecule fluorescence** (Ma et al., 2012)
			**Simulation** (Mincer and Simon, 2011)
**Selective phase/hydrogel**	No	No	**Biochemical assays** (Frey and Görlich, 2007; Labokha et al., 2013)
**Polymer brush**	No	No	**Immunogold electron microscopy/AFM** (Lim et al., 2007)
			**Simulation** (Miao and Schulten, 2010)
**ROD**	No	Yes	**Optical trapping/single-molecule tracking** (Schleicher et al., 2014)
**Forest**	Yes	Yes	**AFM** (Liashkovich et al., 2012)
			**Cryo-ET** (Eibauer et al., 2015)
			**Simulation** (Ando and Gopinathan, 2017)
**Dilation**	Yes	Yes	**AFM** (Kastrup et al., 2006)
			**Cryo-EM** (Zimmerli et al., 2021)
			**SM fluorescence** (Pulupa et al., 2020)
**Transport receptor**	Yes	Yes	**AFM** (Lim et al., 2007)
			**SPR** (Schoch et al., 2012)
			**SM fluorescence** (Ma et al., 2012)

Advancements in imaging techniques offer a new perspective on tackling the challenges posed by the dynamic nature of the NPC. While near-atomic resolution has been achieved, these static imaging approaches have limitations in capturing the dynamic nature of the NPC. The application of super-resolution microscopy techniques, capable of providing high spatiotemporal resolution within live cells, holds promise for exploring the dynamic behavior of the NPC. Such approaches could allow researchers to observe the real-time changes in the conformation and interactions of FG-Nups, NTRs, and cargo molecules, providing a more accurate representation of the NPC’s structure and function. By focusing high-spatiotemporal resolution super-resolution methodologies on the IDR FG-Nups in live cells, it is possible that a new model of NPC structure and function may emerge to bridge the discrepancies observed between the current models. Furthermore, the influence of the cellular milieu on the NPC cannot be overlooked. The behavior and structure of the NPC appear to be influenced by the surrounding cellular components and conditions. Investigating the NPC within the context of live cells and considering the interplay between NTRs, cargoes, and FG-Nups will provide valuable insights. By examining the dynamic interactions among these components, researchers may uncover key mechanisms that contribute to the NPC’s functionality.

Overall, understanding the complexity of the NPC requires incorporating multiple perspectives and employing advanced imaging techniques capable of capturing its dynamic nature within live cells. By addressing the limitations of current models and considering the intricate interplay between the NPC and its cellular environment, researchers can make significant strides toward understanding the complexities of this essential cellular structure. Such advancements will pave the way for developing a comprehensive model that accurately describes the structure and behavior of the NPC.

In conclusion, we endeavor to accentuate the complexity inherent in appraising the profusion of proposed models. Each model presents distinct merits within delineated domains. Concomitantly, it is imperative to acknowledge the absence of irrefutable corroboration for any of these models, as substantiated by empirical evidence. Furthermore, these models often engender incongruent viewpoints and divergent proposals. Our earnest apologies are extended for any unintended misconstruals or inadequate acknowledgments accorded to specific models. Moreover, we express regret that spatial constraints have impeded the inclusion of contributions from select colleagues within this review.
